# Extralesional microvascular and structural macular abnormalities in cytomegalovirus retinitis

**DOI:** 10.1038/s41598-020-78587-6

**Published:** 2020-12-08

**Authors:** Nida Wongchaisuwat, Sansanee Khongpipatchaisiri, Sutasinee Boonsopon, Pitipol Choopong, Nattaporn Tesavibul, Supalert Prakhunhungsit, Somanus Thoongsuwan, Nuttawut Rodanant, Nopasak Phasukkijwatana

**Affiliations:** Department of Ophthalmology, Faculty of Medicine Siriraj Hospital, Mahidol University, 2 Wang-Lang Road, Siriraj Hospital, Bangkoknoi, Bangkok, 10700 Thailand

**Keywords:** Outcomes research, Prognostic markers

## Abstract

To evaluate extralesional microvascular and structural changes of the macula using optical coherence tomography angiography (OCTA) and structural OCT in cytomegalovirus retinitis (CMVR). An observational study of CMVR patients were performed. Complete ophthalmic examination, serial color fundus photography, structural OCT and OCTA were performed at baseline and follow-up visits for up to 12 months. The structural OCT was analyzed to evaluate macular areas within, bordering and beyond the CMVR lesions. Extralesional retinal capillary plexus of the macula were evaluated by OCT angiography and compared with the unaffected fellow eyes. Thirteen eyes from 13 patients were enrolled. At baseline, macular areas without CMVR lesions showed decreased vessel density (VD) of both the superficial (P = 0.0002) and deep (P < 0.0001) retinal capillary plexus in eyes with CMVR as compared with the corresponding macular areas of the unaffected fellow eyes. The decrease of VD persisted through the follow-up period for up to 12 months after adjusting for degree of vitreous haze. Structural macular OCT characteristics at the borders and beyond the lesions included intraretinal hyperreflective dots, cystoid macular edema, subretinal fluid and selective ellipsoid zone (EZ) loss. The selective EZ loss found in 6 of 12 eyes showed recovery in 4 eyes after receiving anti-viral treatment. In CMVR eyes, there were microvascular and microstructural abnormalities in the macular area without clinically visible CMVR lesions. Our results provided interesting insights into CMV infection of the retina.

## Introduction

Cytomegalovirus retinitis (CMVR) is the most common ocular opportunistic infection causing visual loss in patients with human immunodeficiency virus (HIV) infection^1–3^. Current advances of various immunosuppressive agents has also led to increased incidence of CMVR in non-HIV patients who are immunosuppressed^4^.

The patho-mechanism of retinal infection in CMVR is largely unknown. Following hematogenous spread of the virus, involvement of pericytes and vascular endothelial cells of the inner blood retinal barrier has been suggested as a critical step of CMV infecting the retina in in vitro*, *ex vivo and post-mortem studies^5–7^. Clinically, occlusive retinal vasculitis is evident and association of CMVR with several ischemic retinal vascular diseases was also reported^8–10^. These pieces of evidence suggest that there could be a global dysfunction of the inner blood retinal barrier or retinal capillary plexus in eyes affected with CMVR. However, there is still a lack of in vivo studies of the retinal capillary plexus in these eyes.

Optical coherence tomography angiography (OCTA) is a recent technology that allows depth-resolved visualization of microvascular blood flow of the macula noninvasively. This opens the opportunity for an in vivo study of the retinal capillary plexus in CMVR eyes in order to provide more understanding of the disease and subsequent therapeutic intervention.

Therefore, this study aimed to investigate microvascular abnormalities in the macula of eyes with CMVR using OCTA. Microstructural changes of the macula were also investigated using structural optical coherence tomography (OCT).

## Methods

This observational study was conducted according to the tenets of the Declaration of Helsinki and the study protocol was approved by the Siriraj Institutional Review Board (IRB), Faculty of Medicine Siriraj Hospital, Mahidol University, Bangkok, Thailand (approval number 258/2561). The study protocol was mainly prospective but data from retrospective patients who met the inclusion and exclusion criteria were also allowed to be collected. A written informed consent was obtained from all prospective participants. Informed consents for retrospective patients were waived by IRB.

## Subjects

Consecutive patients were recruited from the CMVR clinic, Department of Ophthalmology, Faculty of Medicine Siriraj Hospital, Mahidol University, Bangkok, Thailand, between June 2018–September 2019. Inclusion criterion was patients of at least 18 years of age with clinical diagnosis of CMVR, which was based on typical appearance of the disease consisting of areas of retinal opacification or retinitis, variable amount of retinal hemorrhage and inflammatory vascular sheathing. The diagnosis was confirmed by intraocular fluid polymerase chain reaction (PCR) for CMV in cases with controversial clinical findings.

Exclusion criteria included pregnancy; preexisting uveitis apart from CMVR; retinal detachment; maculopathies from other causes such as diabetic retinopathy, retinal vein occlusion or retinal artery occlusion; ocular media opacity prohibiting high quality retinal imaging; inability to cooperate in acquiring good quality retinal imaging. Patients with a history of intraocular surgery in the past 6 months or a history of medication known to affect the macula such as chloroquine, hydroxychloroquine, tamoxifen, taxanes, etc. were also excluded.

During the course of CMVR treatment, patients were followed for clinical examination and retinal imaging at the baseline, 2-week, 1-month, 2-month, 3-month, 6-month and 12-month visits.

### Clinical examination

Demographic data including age, sex and underlying diseases and onset of CMVR were collected. Complete ophthalmic examination including visual acuity (VA), intraocular pressure, slit-lamp biomicroscopy and dilated fundus examination were performed. Degree of vitreous haze (grade 0.5, 1, 2, 3 and 4)^11^, location of retinitis and extent of retinitis (< 25% or > 25% of total retinal area), involvement of the optic disc, macula and major retinal vessels were noted. The location of retinitis is classified as the following: *zone 1* is confined to 1500 μm from the edge of the optic nerve and 3000 μm from the center of the fovea; *zone 2* resides outside zone 1 but within a circle defined by the ampullae of the vortex veins; and *zone 3* is located outside zone 2 to the ora serrata. The details of CMVR treatment and highly active anti-retroviral therapy (HAART) were also collected. 

### Retinal imaging

Retinal imaging including color fundus photography (Kowa VX-20 retinal camera, Tokyo, Japan), spectral-domain structural OCT (SPECTRALIS^®^, Heidelberg Engineering, Heidelberg, Germany) of the macula and OCTA (RTVue XR Avanti, Optovue, Inc, Fremont, CA) were obtained.

Spectral-domain OCT macular scans were performed to investigate structural changes of the CMVR lesion, the border of the lesion and the macular area beyond the lesion. At least 25 horizontal scans covering at least 20° horizontally and 20° vertically centering at the fovea were performed. Each scan session was registered to the baseline visit. The border of the lesion was defined as 500 μm within and 500 μm outside the visible margin of retinal infiltrate/hemorrhage. The macula was defined as a circular area of 5500 μm in diameter surrounding the fovea.

The OCTA scans were obtained with an A-scan rate of 70,000 scans/s, a light source of 840 nm and a bandwidth of 45 nm. Each scan covered 6 × 6 mm centered on the fovea (6 × 6 mm HD Angio Retina). Superficial and deep retinal capillary plexus were segmented automatically by the RTVue XR Avanti software (version 2017.1 with DualTrac). Segmentation was manually checked in every patient. In case of segmentation errors, the segmentation was manually adjusted to the following: (1) superficial segmentation was bordered by the internal limiting membrane and the junction between inner plexiform layer (IPL) and inner nuclear layer (INL), and (2) deep segmentation was bordered by the IPL-INL junction and the junction between outer plexiform layer (OPL) and outer nuclear layer (ONL). Only Scan Quality (SQ) of at least 6 was accepted for further quantitative analysis as suggested by the manufacturer.

### Quantitative vascular density analysis

Vascular density (VD) defined as the percentage of the sample area occupied by vessel lumens was automatically calculated by the built-in AngioAnalytics software in the OCTA machine.

To address our hypothesis of global retinal microvascular involvement in eyes with CMVR, we studied the VD of macular capillary plexus in the area without visible CMVR lesion. It was not possible to study capillary plexus within the lesion itself due to tissue necrosis and total disorganization of retinal structures. The corresponding macular area in the unaffected fellow eye was chosen for VD comparison. Patients with bilateral CMVR or one-eyed patients were excluded from the VD analysis due to a lack of comparative unaffected eyes.

In the AngioAnalytics software, the 6 × 6 mm macular area was divided into several sectors based on Early Treatment Diabetic Retinopathy Study (ETDRS) grid, which consisted of 3 circles of 1, 3 and 6 mm centered on the fovea. The foveal center was automatically determined by the software but was manually rechecked and adjusted in every OCTA image for accuracy. The ring areas between each circle were subdivided into superior, inferior, temporal and nasal sectors. Alternatively, the macula was equally divided into 9 grid sectors corresponding to 9 squares within the 6 × 6 mm area. The software automatically calculated the VD in each of these sectors. In an affected eye, VD of the sectors without visible CMVR lesion were combined and compared with the corresponding sectors in the unaffected fellow eye as mentioned above. When the CMVR lesion was totally outside the macula, the whole area of the ETDRS grid was used for VD analysis. Sectors with significant blockage by vitreous floaters were not included in VD analysis in both affected and unaffected eyes.

### Statistical analysis

Categorical data were presented as numbers and percentages. Continuous data were presented as mean and standard deviation (SD). Since for each patient, VD was measured at 2–7 different time points, mixed models were fitted to test the differences in VD between the affected and unaffected eyes after adjusting for vitreous haze. SAS Studio 9.2 and PASW 18.0 were used for statistical analyses. A P-value of less than 0.05 was considered statistically significance.

## Results

Twenty-three CMVR patients were identified in our CMVR clinic from June 2018 to December 2019 and met the inclusion criteria. Ten patients were excluded due to retinal detachment (6 patients) and negative PCR for CMV (4 patients). The final remaining 13 patients were recruited in the study. Data collection was performed prospectively in 11 patients and retrospective review of available data was done in 2 patients. Demographic characteristics of all participants were shown in Table 1.

**Table 1:** Demographic characteristics of study participants.

No	Age	Log MAR VA	Laterality	Underlying condition	Onset of CMVR	Vitreous haze	Classification	Location of lesion	Macula involvement	Systemic treatment	No. Of IVT	Follow up time	Time to quiescence
1	51	0.1	Unilateral	Lymphoma	At screening	1	Necrotizing	Zone 2	No	Cidofovir	4	1 year	3 month
2	72	0.5	Unilateral	Refractory MM	1 month	0	Necrotizing	Zone 1-2	Yes	Gangciclovir	> 50	1 year	Still active
3	37	0	Unilateral	Lymphoma	1 week	1	Necrotizing	Zone 1-3	Yes	Gangciclovir	8	1 year	9 month
4	24	0.3	Unilateral	HIV (CD4 = 108)	1 month	1	Necrotizing	Zone 2-3	No	Gangciclovir	1	1 month	Still active, RRD
5	33	0.1	Unilateral	HIV (CD4 = 39)	At screening	0	Necrotizing	Zone 2-3	No	Oral valgangciclovir	0	10 month	6 month
6	32	0.5	Bilateral	HIV (CD4 = 15)	1 month	0	Necrotizing	Zone 2-3	No	Gangciclovir	6	1 year	4 month
7	84	0.3	Unilateral	Lymphoma, POAG	1 month	1	Necrotizing	Zone 1-3	Yes	Gangciclovir	> 50	1 year	Still active
8	19	0.5	Unilateral	HIV (CD4 = 139)	1 month	0	Necrotizing	Zone 1-2	Yes	Gangciclovir	11	1 year	6 month
9	35	0.5	Unilateral	HIV (CD4 = 122)	3 month	0.5	Necrotizing	Zone 2	No	No	9	8 month	Still active
10	41	0.2	Unilateral	SLE	2 month	0	Necrotizing	Zone 2-3	No	Ganciclovir	10	8 month	Still active
11	43	0	Unilateral	HIV (CD4 = 21)	At screening	0	Necrotizing	Zone 3	No	Ganciclovir	0	6 month	1 month
12	25	0.6	Unilateral	Polymyositis	2 week	0	Frosted branch	Zone 1-2	Yes	Ganciclovir	18	9 month	Still active
13	60	0	Bilateral	SLE	1 week	0	Necrotizing	Zone 2-3	No	No	20	8 month	Still active

*CMVR* cytomegalovirus retinitis, *HIV* human immunodeficiency virus, *IVT* intravitreal ganciclovir injection, *MM* multiple myeloma, *POAG* primary open angle glaucoma, *RRD* rhegmatogenous retinal detachment, *SLE* systemic lupus erythematosus, *VA* visual acuity.

Twelve patients showed classic necrotizing retinitis and 1 patient demonstrated frosted branch angiitis at the initial presentation. Among the former, 4/12 (33%) patients had retinitis lesions involving the macular area, while 8/12 (67%) patients had lesions outside the macular which could not be observed by the macular OCT scans.

### Microstructural changes of the macula in CMVR

Structural OCT of the retinitis lesion could be studied in 4 eyes which had retinitis within the macular area and all eyes exhibited full thickness hyperreflective retinal thickening followed by retinal thinning and atrophy after treatment. Two eyes progressed to full thickness retinal disruption with overlying vitreoretinal gliosis.

**Figure 1:**
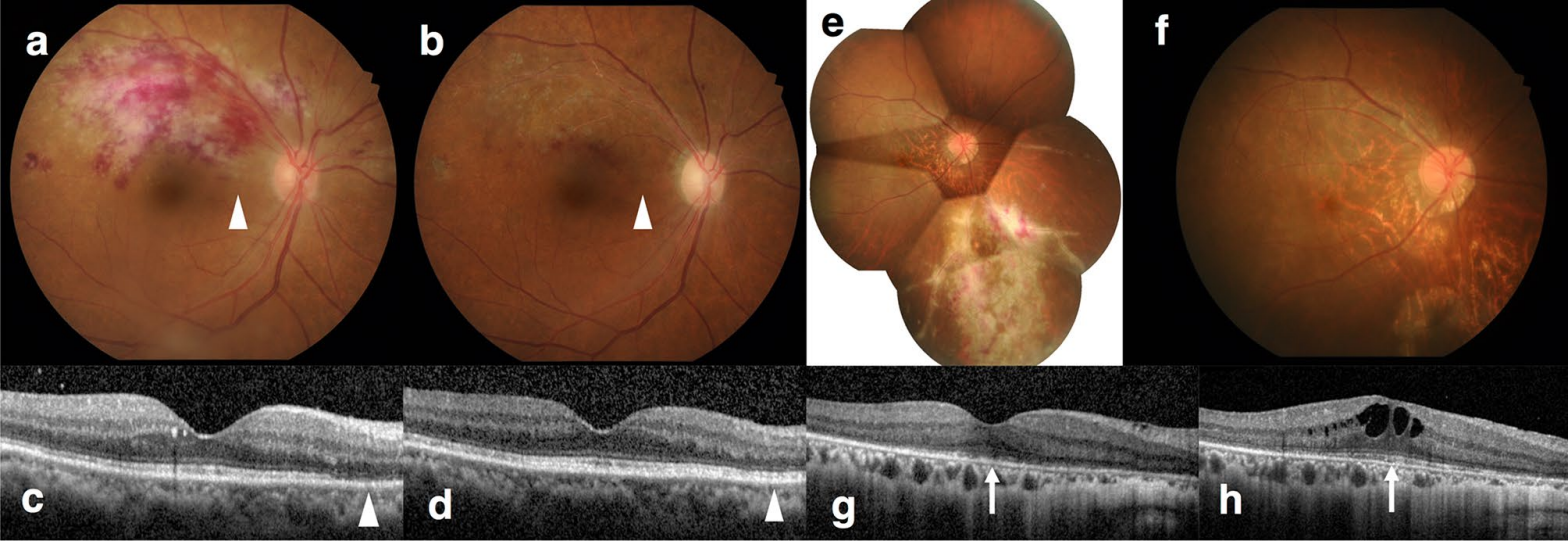
Recovery of ellipsoid zone (EZ) disruption in cytomegalovirus retinitis (CMVR) Patient No.2 (**a**–**d**). Fundus photograph (**a**) shows retinal infiltrates and intraretinal hemorrhages at the superotemporal quadrant involving the macula. The corresponding optical coherence tomography (OCT) scan (**c**) at the level of fovea demonstrates loss of EZ at the border of the active lesion (arrowheads in **a** and **c**). After treatment, the lesion subsided (**b**) with recovery of EZ on the registered OCT scan (**d**) at the exact location (arrowheads in **b** and **d**). Patient No. 6 (**e**–**h**). Fundus photograph (**e**) and macular OCT (**g**) of the right eye with an active CMVR lesion show extralesional EZ loss at the macula (arrow in **g**). Partial recovery of EZ at the center of fovea was observed after therapy (arrow in **h**) with some cystoid macular edema and mild pigmentary changes of the macula on the fundus photograph (**f**).

Structural macular OCT characteristics at the lesion boundaries and beyond the lesions were studied in 8 eyes and consisted of selective ellipsoid zone (EZ) disruption, inner retina opacification with EZ sparing, intraretinal hyperreflective dots (mostly in the INL), cystoid macular edema (CME) and subretinal fluid (SRF). These macular findings, except for inner retina opacification, were found even in eyes with retinitis lesions located totally away from the macula. These structural OCT characteristics were summarized in Table 2. Interestingly, 4 of 6 eyes (67%) with selective EZ disruption exhibited partial or complete recovery of the EZ during the course of antiviral treatment (Fig. 1).

**Table 2:** Structural optical coherence tomography (OCT) findings of the macula in cytomegalovirus retinitis patients at the retinitis lesions, borders of the lesions and extralesional macular areas.

Macular OCT characteristics	No. of eyes	
	Eye with macular lesion (n = 4)	Eye without macular lesion (n = 8)
**Intralesion**		
Full thickness hyperreflective retinal lesion	4/4	NA
Retinal thinning /atrophy	4/4	NA
Retinal pigment epithelial atrophy and choroidal hyperreflectivity	4/4	NA
Full thickness retinal disruption	2/4	NA
Vitreoretinal gliosis and traction during follow up	2/4	NA
Epimacular membrane	3/4	NA
Vertical strip of hyperreflectivity in the outer nuclear layer	2/4	NA
Disorganization of retinal inner layers	3/4	NA
**Border or extralesion**^**a**^		
Selective loss of EZ		
Border of lesion	4/4	NA
Recovery of EZ	2/4	NA
Extralesion	0/4	2/8
Recovery of EZ	0/4	2/8
Infected inner retina with spared EZ	1/4	0/8
Hyperreflective dot in retina	4/4	1/8
Cystoid macular edema		
Border of lesion	2/4	NA
Extralesion	NA	1/8
Subretinal fluid		
Border of lesion	2/4	NA
Extralesion	NA	2/8

*EZ* ellipsoid zone, *NA* not applicable.

^a^Border of lesion defined as 500 μm within and 500 μm beyond visible margin of the lesion.

The only patient with frosted branch angiitis, a less common clinical presentation of CMVR, showed white perivenular sheathing. Structural OCT showed hyper-reflective thickening of vessel walls which resolved and returned to normal anatomy after treatment with systemic gangciclovir.

### Microvascular changes of the macula in CMVR

**Figure 2:**
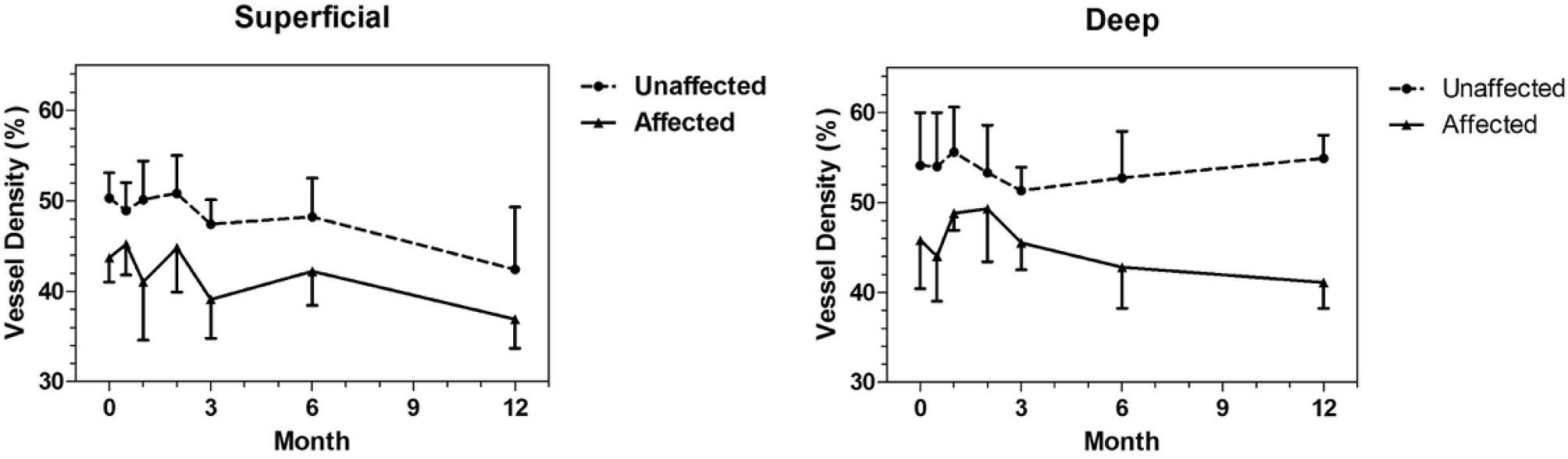
Mean extralesional vascular density of the superficial and deep capillary plexus at each time point between affected and unaffected eyes. Standard deviations were defined by vertical lines.

**Figure 3:**
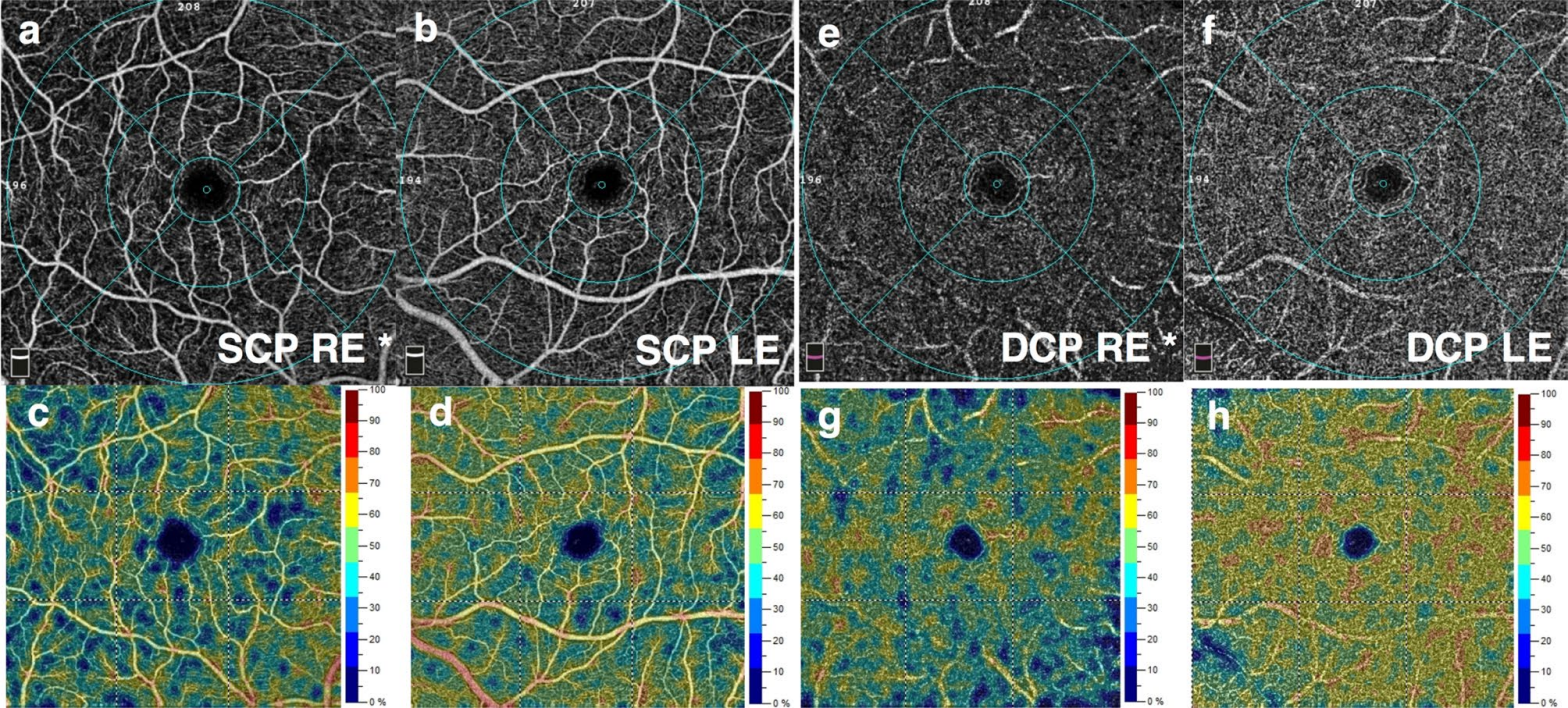
Optical coherence tomography angiograms (top row) and corresponding color mapping of vessel density (bottom row) of the macula of patient No.5 in an eye with CMVR without macular involvement (**a**,**c**,**e**,**g**) and the contralateral normal eye (**b**,**d**,**f**,**h**). Decreased vessel density in the superficial (**a**–**d**) and deep capillary plexus (**e**–**h**) was observed in the affected eye compared with the unaffected fellow eye. Asterisks denote the affected eye. LE, left eye; RE, right eye; DCP, deep capillary plexus; SCP, superficial capillary plexus.

VD analysis by OCTA could be performed in 8 of the 13 patients. Five patients were excluded for the following reasons: 1 patient with poor quality OCTA image, 1 retrospective patient without available OCTA, 2 bilateral CMVR patients, and 1 patient with frosted branch angiitis. At baseline visit, the mean VD in the extralesional macular areas of the affected eyes was significantly less compared with the corresponding macular areas of the unaffected fellow eyes for both the superficial (P = 0.0002) and deep (P < 0.0001) retinal capillary plexus (Table 3). Longitudinal OCTA follow-up during antiviral treatment revealed persisitent decrease of the extralesional macular VD at all time points in the affected eyes compared with the unaffected fellow eyes for up to 12 months (Table 3 and Fig. 2). All the mean differences of VD were tested by mixed models which took into account the dependent nature of data between eyes of the same individuals and repeated measurements with time. The degree of vitreous haze was also used to adjust the mean differences of VD in the models, although it was mild (0–1) in all patients as shown in Table 1. Representative macular OCT angiograms of the SCP and DCP with VD color mapping in a patient with extramacular CMVR (patient No. 5) were presented in Fig. 3.

**Table 3:** Comparison of extralesional vascular density in the superficial and deep capillary plexus between affected and unaffected eyes of unilateral CMVR patients .

Retinal capillary plexus	Month	Vascular density (%)							P-value*
		Unaffected			Affected			Mean difference*	
		n	Mean	SD	n	Mean	SD		
Superficial	0	8	50.3	2.8	8	43.7	2.7	6.43	0.0002
	0.5	5	48.9	3.1	5	45.2	3.4	3.62	0.0600
	1	5	50.1	4.3	4	41	6.4	8.32	0.0003
	2	6	50.8	4.2	5	44.8	4.9	5.4	0.0050
	3	4	47.4	2.7	4	39.1	4.3	8.39	0.0002
	6	5	48.2	4.3	5	42.2	3.8	5.98	0.0023
	12	2	42.4	6.9	3	36.9	3.2	5.38	0.0518
Deep	0	8	54.1	5.9	8	45.8	5.4	9.21	< 0.0001
	0.5	5	54	6	5	44	5	10.78	0.0002
	1	5	55.6	5	4	48.8	1.9	8.25	0.0097
	2	6	53.3	5.3	5	49.3	5.9	4.25	0.1068
	3	4	51.3	2.6	4	45.5	3	5.62	0.0842
	6	5	52.7	5.2	5	42.8	4.6	9.85	0.0005
	12	2	54.9	2.6	3	41.1	2.9	13.91	0.0007

*Mean differences of vascular density between affected and unaffected eyes and P-value derived from mixed model analysis after adjusting for vitreous haze.

There were no obvious morphological microvascular changes or differences in the foveal avascular zone area between the two groups.

## Discussion

This study demonstrated the presence of microvascular and microstructural abnormalities in the macular area without visible CMVR lesions on clinical examination in eyes with CMVR. There was a decrease in the VD of both superficial and deep retinal capillary plexus by quantitative OCTA analyses. The extralesional decrease in VD seemed to persist during the treatment follow-up period for up to 1 year, even though the retinitis had subsided. The microstructural changes including selective loss of EZ, CME and SRF could also be found in the macula beyond the CMVR lesions.

Previous studies have provided some evidence of impaired retinal capillary of the inner blood retinal barrier (IBRB) associated with CMV infection of the retina^6,7,12^. In a post mortem study, retinal capillary plexus was devoid of endothelium in the retinitis lesion and few vessels at the margin of the lesion demonstrated CMV viral proteins in the endothelium^5^. In addition, CMV inclusions in the inner retinal cells and swollen retinal endothelial cells were found in areas peripheral to the CMVR lesions with well-organized retinal structure^5^ indicating the presence of subclinical CMV infection in the extralesional retinal areas. In a cell-culture model of IBRB, pericytes was found to be highly permissive for lytic CMV infection and was suggested to be an amplification reservoir of CMV, leading to retinal inflammation^7^. Our results are supported by those studies. In particular, the decrease in extralesional VD in CMVR eyes found in our study might be explained by subclinical endothelium and pericyte loss secondary to CMV dissemination in the IBRB. On the other hand, damage to the IBRB itself could in turn facilitate CMV infection of the retinal cells^6^. We hypothesized that in patients with CMV, the retinal areas where the impairment of IBRB reached a certain level could subsequently manifest clinical retinitis. To the best of our knowledge, this is the first in vivo study of retinal microvasculature in CMVR. Further studies are still needed to elucidate the role of IBRB in CMVR.

The characteristics of structural OCT of the CMVR lesions observed in this study agreed with previous studies^13–15^. Total retinal hyperreflectivity and thickening followed by progressive retinal thinning and RPE atrophy were observed. Persistent posterior hyaloid attachment, vitreoretinal gliosis and secondary ERM formation can lead to retinal traction and retinal detachment^16^.

Extralesional abnormalities of structural OCT of the macula has hardly been described. They were usually found near the lesion margins. However, we did find some abnormalities located far away from the visible retinitis lesions. These included selective EZ disruption, intraretinal hyperreflective dots, CME and SRF. Interestingly, the selective EZ disruption with preserved inner retinal architecture exhibited complete or partial recovery in 4 of 6 eyes (67%) after treatment. Gupta, et. al., described EZ irregularities in the areas just beyond the leading edge of CMVR lesions (within 200 μm) in 9 eyes. Unlike our study, the EZ irregularities in their study was also associated with variable degrees of retinal architectural disruptions and only 1 eye exhibited normalization of the EZ^13^.

The selective EZ disruption found in our study is in agreement with the hypothesis of CMV infection originating from the choroid. Previous animal studies provided evidence of CMV induced disruption of outer blood retinal barrier (OBRB) and spreading of the infection from the choriocapillaris and RPE to the inner retinal cells possibly through Muller cells^6,12,17^. The selective EZ disruption may represent early CMV infection via this route and could be recovered if the treatment was initiated promptly before viral induced photoreceptor apoptosis^18^. Some cases with persistent loss of outer layer with preserved inner retinal layers were described^19^. Moreover, evidence of delineate line of hyperautofluorescence along the active leading edge of the retinitis also suggested early involvement at the RPE/photoreceptor level^20,21^.

As a result, our in vivo study of extralesional microvascular OCTA and microstructural OCT provided evidence supporting that CMV could infect the retina in two manners. Firstly, the infection might initiate from the retinal vessels (IBRB) in the inner retina and then spread downward to the RPE. Secondly, the choroid and RPE (OBRB) might alternatively be the first target infected by the virus which spread in the opposite direction to the former. Invernizzi, et al., described two patterns of structural OCT of CMVR lesions: (1) carvernous necrosis with intact choriocapillaris and RPE, and (2) full-thickness retinitis with RPE and choriocapillaris involvement^14^. Their findings also suggested the two modes of CMV infecting the retina.

Although it is used worldwide, there has been no standard regimen for intravitreal ganciclovir treatment in CMVR. The optimal regimen should balance between efficacy, treatment burden and potential retinal toxicity from prolonged and repeated injections^22–24^. The regimen used in this study has been demonstrated to be clinically effective and safe^25^. Nevertheless, no studies have evaluated the effects of intravitreal ganciclovir on microvascular retinal blood flow. There were a few case reports describing ischemic retinal events after intravitreal ganciclovir injections^26,27^. In our study, the decrease in VD found in CMVR eyes at baseline occurred before treatment and it remained stable through the follow-up period with multiple intravitreal ganciclovir injections in between (Fig. 2 and Table 3) This indicated that our regimen of intravitreal ganciclovir injection did not have significant effects on the VD.

This is a pilot study and there are some limitations. The sample size was small as the disease is relatively uncommon and several patients were excluded due to retinal detachment. The macular area evaluable by OCTA was small. Only 6 × 6 mm central area was assessed and our results may not represent the pathology for the whole retina. CMVR patients were usually immunocompromised and other coexisting systemic diseases might have effects on the retinal microvasculature and VD analysis. However, we performed the analysis comparing between the CMVR eyes and the unaffected fellow eyes of the same individuals, which eliminated confounding effects of the coexisting systemic diseases.

OCTA artefacts could have affected our VD asnalysis. Concerning this, we employed careful strategies to obtain good quality images including SQ threshold of at least 6, meticulous adjustment of segmentation errors and avoidance of areas with significant shadowing by vitreous floaters. Vitreous haze could also affect our results. However, all included patients showed only mild vitritis with vitreous haze grade 0 to 1. Significant differences between mean VD of the affected and unaffected fellow eyes were still observed either with or without (results not shown) adjusting for vitreous haze, indicating low effects of vitreous haze on our results. Moreover, the decrease in VD remained persist during treatment with improvement of the CMVR lesions and clearing of vitritis.

Repeatability and reproducibility of OCTA VD measurements were found to be high but variability of the measurements should also be taken into consideration when interpreting the result^28,29^. More studies with larger sample size and wider field of OCT/OCTA are needed to confirm and further explore our findings.

In summary, this study demonstrated a decrease of macular capillary VD in eyes with CMVR wherever the lesions were located. Extralesional microstructural macular abnormalities could also be detected in these eyes. Our results suggested that these patients might have subnormal visual functions even though the visual acuity was normal. Further studies of other visual functions such as contrast sensitivity and microperimetry in these eyes would be useful. This study also provided interesting insights into CMV infection of the retina, which would lead to better understanding and subsequent therapeutic improvement of this devastating disease.
